# Enhanced Pulsatile Growth Hormone Secretion and Altered Metabolic Hormones by in Vivo Hexarelin Treatment in Streptozotocin-Induced Diabetic Rats

**DOI:** 10.3390/ijms19103067

**Published:** 2018-10-08

**Authors:** Xinli Zhang, Jin-Kui Yang, Chen Chen

**Affiliations:** 1 School of Biomedical Sciences, University of Queensland, St Lucia, Brisbane, QLD 4072, Australia; cesc0821@hotmail.com; 2 School of Medicine, Faculty of Medicine, Capital Medical University, Beijing 100730, China; jinkui.yang@foxmail.com

**Keywords:** growth hormone, streptozotocin (STZ), hexarelin, IGF-1, free fatty acids, glucose

## Abstract

Significant growth hormone (GH) reductions have been reported in diabetic animal models with disturbed metabolic balance coinciding with GH deficiency. Therefore, enhanced GH secretion may have beneficial effects in controlling diabetes. Thus, we aim to investigate the effect of hexarelin, a synthetic GH secretagogue (GHS), on GH secretion in streptozotocin (STZ, 65 mg/kg)-induced diabetic rats. Daily hexarelin (100 μg/kg) treatment was performed for two weeks in four-week-long STZ-diabetic and vehicle control rats. Pulsatile GH secretion in STZ-rats was significantly reduced in total, pulsatile, basal, and mass of GH secretion per burst. In addition, impaired GH secretion was followed by an increase in fasting-level free fatty acids (FFAs) and a decrease in insulin-like growth factor 1 (IGF-1) compared to control rats. After hexarelin treatment, pulsatile GH secretion in STZ-rats was significantly increased in total, pulsatile, and basal, but not in the mass GH secretion per burst, compared to STZ-rats without hexarelin treatment. However, there was no significant elevation in GH secretion in the hexarelin-treated control group. In addition, hexarelin-treated STZ-rats showed a significant decrease in fasting level FFAs, whereas suppression of fasting level for IGF-1 was maintained. These results suggest that STZ-induced diabetic rats have impaired pulsatile GH secretion, causing increased FFAs and decreased IGF-1 levels in circulation. Hexarelin injections for two weeks is able to normalize impaired pulsatile GH secretion with normal fasting levels of FFAs, but fails to recover IGF-1 levels.

## 1. Introduction

Growth hormone (GH) is synthesized, stored, and secreted by somatotrophs in the anterior pituitary gland. It performs an anabolic role in modulating metabolic balance in the body. Hypothalamic growth hormone-releasing hormone (GHRH) is a major regulator of GH secretion, which stimulates pituitary GH release; whereas somatotropin-releasing inhibiting factor (SRIF) or somatostatin neurons plays an inhibitory effect on pituitary GH release [1,2]. Insulin-like growth factor 1 (IGF-1) release is predominantly regulated by GH in various tissues, particularly in the liver because more than 50% of circulating IGF-1 is originally secreted from hepatic tissue [3]. The GH/IGF-1 axis exerts a significant role in cell and tissue function because both hormones stimulate tissue growth and metabolic activities [4]. GH is inversely related to glucose levels with an increase in GH under the hypoglycemic condition. Elevated GH levels in peripheral tissues suppress glucose oxidation with increased liver gluconeogenesis, lipolysis and circulating fasting-level free fatty acids (FFAs) [5,6]. Most experimental models showed that depressed IGF-1 levels were correlated with decreased GH levels [7,8,9]. GH release was inhibited in diabetic animal models, which was featured by decreased basal GH and lack of GH pulsatility, possibly caused by a reduction in GH synthesis, storage, and stimulated secretion [10,11,12]. A study on streptozotocin (STZ)-induced diabetic rats also showed reduced GH pulsatility and SRIF antiserum abolished such suppressed GH profiles [9]. Depressed GH concentration in the diabetic model was associated with elevated SRIF levels and a reduction of GH response to GHRH [7,13,14,15]. Diabetic rat studies also exhibited declined hypothalamic GHRH secretion [2,12,13]. Depressed GH receptor expression might be another contributor to GH deficiency in the diabetic models [16]. Insulin appeared to perform a role through particular receptor regulation, including the GH receptor and hepatic glucagon receptors [17]. Experimental diabetes studies showed that insulin deficiency resulted in decreased expression of GH receptors, which was restored by insulin treatment with normalized level of IGF-1 [16]. Growth hormone secretagogues (GHS), including hexarelin (Hex, a peptide synthetic GHS), have been shown to stimulate GH secretion in several species of animals, and also in humans, while a certain level of GHRH is considered as the essential mediator of GHS-stimulated GH release [18,19]. It has been reported that GHRH antiserum or GHRH receptor (GHRH-R) antagonists may cause depressed GH secretion to GHS (GHRP-6) injection [20,21]. Clinical studies also showed suppressed GH response to administration of GHS in GHRH-R deficient patients [22,23]. Additionally, increased GH release was associated with elevated GHRH-R expression after long-term Hex treatment, suggesting that GHSs may stimulate GH secretion through the action of GHRH [24]. Both in vivo and in vitro investigations demonstrated that arcuate nucleus (ARC) c-fos expression was increased by the GHS-stimulating effect, as GHS-R expression was present in ARC GHRH neurons [25,26,27]. GHRP-6 treatment in pigs showed that elevated GH release was correlated with c-fos activation in the pituitary, but not the hypothalamus, suggesting that the GHS-mediated GH release effect primarily occurs in the pituitary [28]. Thus, we aim to investigate whether Hex is capable of ameliorating GH secretion in STZ-induced diabetic rats with significant a GH reduction. The beneficial effects of this increase in GH will also be studied.

## 2. Results

### 2.1. Pulsatile GH Secretion in STZ- and Hex-Treated Rats

**Figure 1:**
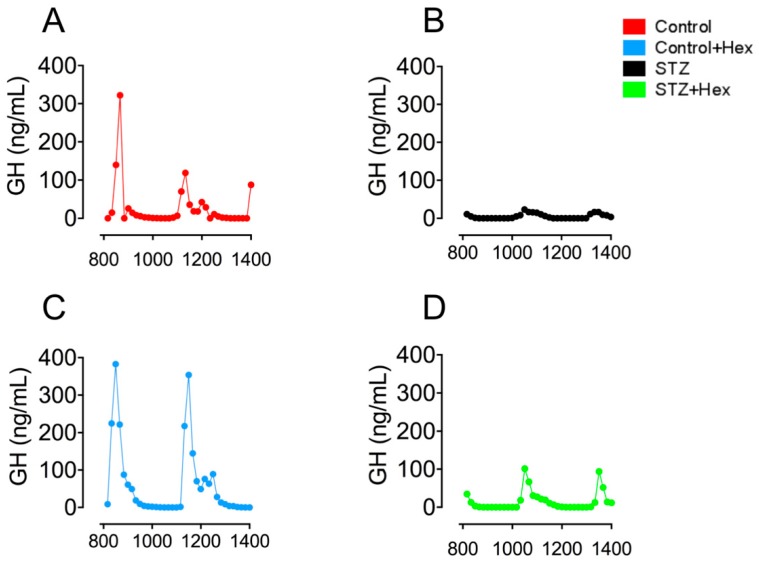
Pulsatile GH secretion from control, control + Hex, STZ, and STZ + Hex rats. Representative profiles of pulsatile GH secretion from (**A**) control, (**B**) STZ, (**C**) control + Hex, and (**D**) STZ + Hex rats. *n* = 16.

**Figure 2:**
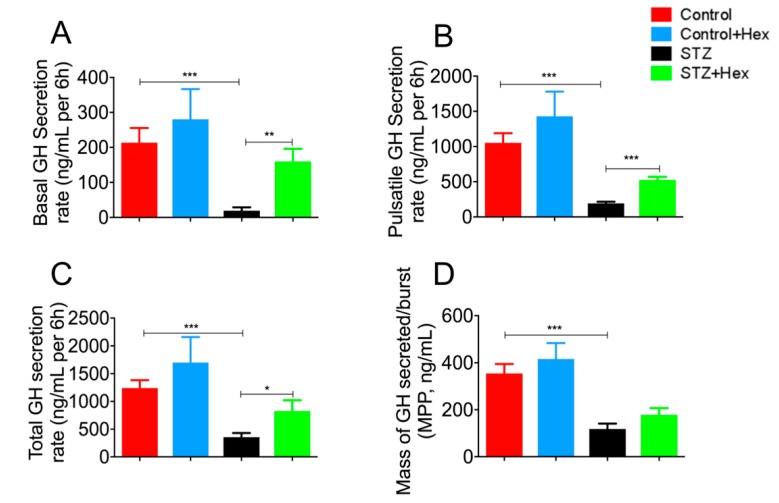
Deconvolutional analysis of pulsatile GH secretion from control, control + Hex, STZ, and STZ + Hex rats. (**A**) Basal, (**B**) pulsatile, (**C**) total, and (**D**) the mass of secreted per burst were determined by deconvolutional analysis. *n* = 16, data are shown as the mean ± SEM. * *p* < 0.05, ** *p* < 0.01, *** *p* < 0.001.

Results (Figure 1) exhibited that declined pulsatile GH secretion peak levels were associated with decreased pulse numbers in STZ-diabetic rats. Hex administration increased the level of pulsatile GH secretion in both the control and STZ groups. Subsequent deconvolution analyses showed that GH secretion in STZ-treated animals was featured by a dramatical decrease in basal (19.2 ± 9.67 vs. 213 ± 41.9 ng/mL per 6 h, *p* < 0.001), pulsatile (192 ± 24.6 vs. 1053 ± 136 ng/mL per 6 h, *p* < 0.001), total (356 ± 75.3 vs. 1243 ± 141 ng/mL per 6 h, *p* < 0.001), and in the mass of GH secreted per burst (118 ± 23.0 vs. 355 ± 39.9 ng/mL, *p* < 0.001), compared to controls (Figure 2). Hex treatment recovered GH release in STZ-diabetic animals significantly in total (826 ± 197 vs. 356 ± 75.3 ng/mL per 6 h, *p* < 0.05), pulsatile (524 ± 44.6 vs. 192 ± 24.6 ng/mL per 6 h, *p* < 0.001), and basal (159 ± 36.6 vs. 19.2 ± 9.67 ng/mL per 6 h, *p* < 0.01), but not in the mass of GH secreted per burst (178 ± 28.9 vs. 118 ± 23.0 ng/mL, *p* = 0.14) (Figure 2). Although GH secretion was increased after Hex treatment, the value was not statistically significant.

### 2.2. Glucose Tolerance Test (GTT) and Insulin Tolerance Test (ITT) in STZ- and Hex-Treated Rats

**Figure 3:**
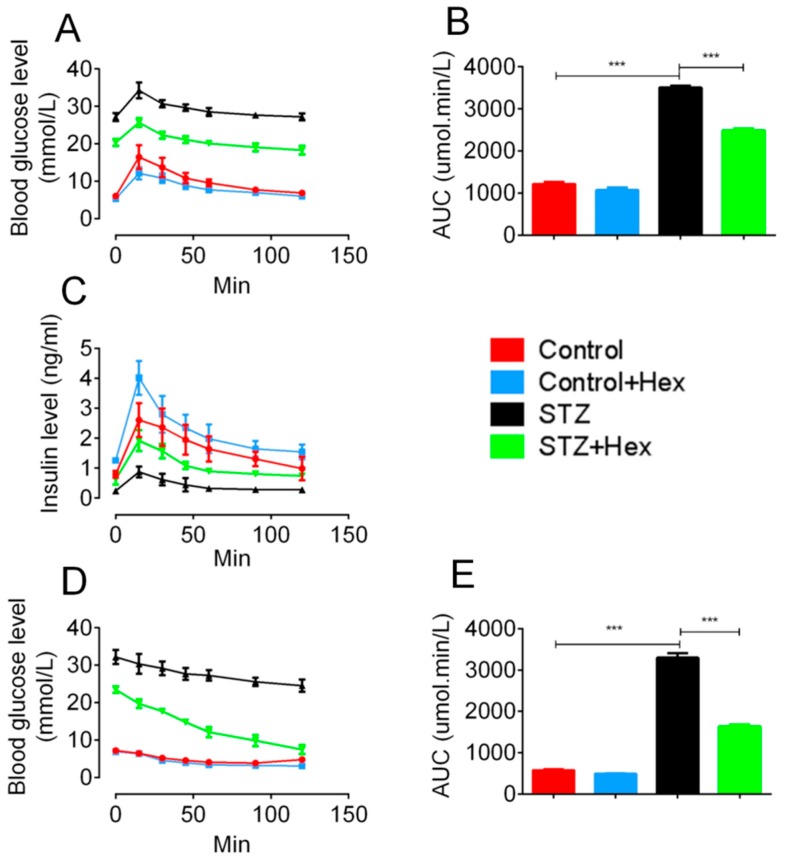
GTT and ITT in control, control + Hex, STZ, and STZ + Hex rats. Glucose tolerance tests and insulin tolerance tests were performed in rats with STZ and/or Hex treatment. Results of (**A**) GTT, (**C**) GTT insulin, and (**D**) ITT are shown at different time points. Corresponding analysis of the area under curve of (**B**) GTT and (**E**) ITT exhibited glucose intolerance and insulin insensitivity of STZ group, which recovered by Hex treatment. *n* = 8, data are shown as the mean ± SEM. *** *p* < 0.001.

As shown in Figure 3, Hex-treated STZ animals showed decreased GTT with similar patterns in comparison with those in the STZ group. Rats in the control group exhibited higher blood glucose levels at 5 and 30 min after glucose injection, compared to Hex-treated controls. Area under curve analysis (Figure 3B) showed a significant elevation of blood glucose levels in STZ rats, suggesting decreased insulin response to glucose in the STZ animals. Hex treatment partially restored impaired glucose response in the STZ group. Consistent with GTT, the levels of insulin secretion in STZ animals were significantly suppressed, whereas both the Hex-treated control and STZ groups showed an increase in glucose-stimulated insulin secretion. 

Three days after GTT, ITT was conducted to determine insulin sensitivity (Figure 3C). Following six hours of fasting, the experiment was performed from 2:00 pm in the afternoon. A total of 1 IU/kg human recombinant insulin was intraperitoneally injected into animals after measurement of blood glucose level at the 0 point. Examination of blood glucose was then conducted at 15, 30, 45, 60, 90, and 120 min after the insulin injection. The STZ group shared the similar pattern of glucose change in response to insulin administration with those of the controls, though blood glucose levels were still maintained at a high level in the STZ group. A declined blood glucose level was observed at 90 min after insulin injection, suggesting elevated insulin sensitivity in Hex-treated STZ rats, but not in normal control rats.

### 2.3. Circulating Levels of IGF-1 and FFAs in STZ- and Hex-Treated Rats

**Figure 4:**
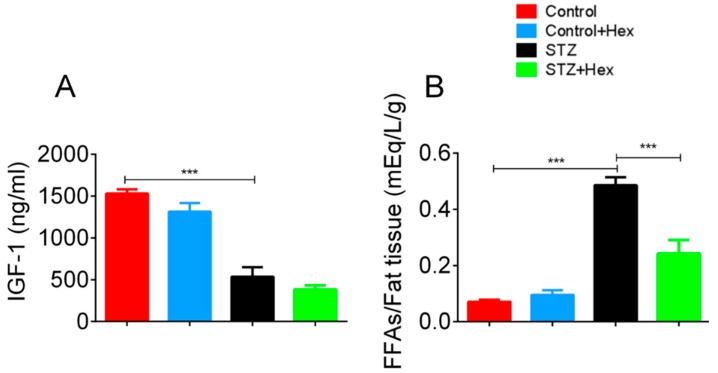
Circulating levels of IGF-1 and FFAs from control, control + Hex, STZ, and STZ + Hex rats. After the STZ or Hex treatment, terminal blood samples were collected. Circulating levels of (**A**) IGF-1 and (**B**) FFAs relative to fat tissue were determined by IGF-1 and FFAs ELISA kits. *n* = 6 from each group, data are shown as the mean ± SEM. *** *p* < 0.001.

Commercial assay kits were employed to determine circulating levels of IGF-1 and FFAs. The plasma IGF-1 level was dramatically declined in the STZ group, while a five-fold increase was shown in FFA levels relative to the amount of fat tissue (Figure 4). Hex treatment normalized the elevated FFA levels, but did not change plasma IGF-1 levels, indicating that circulating FFAs might be transported into fat tissue, facilitated by Hex.

## 3. Discussion

Change of GH secretion in diabetes remains controversial between animal and human studies. Increased GH release was observed in Biobred (BB) rats and Type 1 diabetic patients [29,30,31]. However, the present study exhibited depressed pulsatile GH secretion in STZ-induced diabetic rats, consistent with previous reports on similar STZ rat models [9,11,13]. Studies on STZ-induced diabetic animals have shown that decreased basal and pulsatile GH secretion was associated with reduced circulating IGF-1, pituitary GH mRNA expression, and expression of hypothalamic GH-releasing hormone (GHRH) [2,32]. Depressed GH in STZ-induced diabetic rats is possibly caused by elevated levels of hypothalamic somatostatin (SS). It has been demonstrated that hypothalamic SS tone was increased with decreased GH and GHRH secretion in a single high dosage in STZ-treated rats [1,2,33,34]. However, Park’s group found that the GH axis responded variably in different STZ regime [34]. They demonstrated that multiple low dosages of STZ-injected rats showed normal GH axis activity, except for decreased IGF-1 levels, whereas one single high dosage in STZ-treated rats reduced GH with decreased plasma IGF-1, as well as decreased gene expression of pituitary GH and hypothalamic GHRH. Hence, hyperglycemia may not be the primary modulator of GH secretion in STZ-treated diabetes. The reason why variable STZ regimes caused differential responses of the GH axis remains unknown. As the present study also used a single dose of STZ to induce diabetes, it was believed that depressed secretion of pulsatile GH in the STZ-treated group was a result of decreased hypothalamic GHRH activity with increased somatostatin tone. GHSs are able to stimulate GH secretion in both STZ-induced mice and rats. Dose-dependent GH responses have been observed in both control and STZ rats with GHRH and GHRP-6 stimulations [35]. Elevated GH response in STZ-treated rats was induced by a high dosage GHRP-6 (30 μg/kg) injection, whereas low dose GHRP-6 (3 μg/kg) or administration using only GHRH showed no difference of GH release between STZ or control rats [35]. A potent and similar GH secretion was observed in both control and STZ groups when receiving a combined injection of GHRH and GHRP-6. Anti-GHRH serum in passive immunization eliminated the effect of GHRP-6 on GH responses, indicating GHRP-6 or GHS may stimulate GH secretion in presence of GHRH from hypothalamus [24,36]. Similar effects on GH secretion were also observed by other GHS treatments, such as ghrelin and ipamorelin, in both control and STZ-induced diabetic rodents [37,38]. Therefore, as a member of the GHS family, Hex may exert the same effect on pulsatile GH secretion in both control and STZ-treated rats.

Glucose intolerance occurred in STZ-treated rats due to STZ-induced toxic effect on islet β-cells. GTT study on STZ-treated mice demonstrated that high blood glucose levels remained during the experiment, indicating impaired glucose-stimulated insulin secretion [39]. As Hex protected β-cells from STZ toxic effects [40], high blood glucose level in STZ-Hex co-treated rats was ameliorated in this study, possibly through a restored function of glucose-induced insulin release. Furthermore, results of ITT exhibited that the same pattern of insulin response was found in both control and STZ groups, indicating STZ did not change insulin sensitivity. However, elevated insulin sensitivity was observed in Hex-treated STZ animals but not in Hex-treated control animals. Because there are no studies reporting how Hex increases insulin sensitivity, the possible mechanism may include peroxisome proliferator-activated receptors isoform γ (PPARγ), a metabolic regulator of insulin sensitivity. The thiazolidinediones (TZDs), a high-affinity ligand for PPARγ, is able to enhance insulin sensitivity through PPARγ activation [41,42,43]. Hex has been shown to promote PPARγ activation [44] which may increase insulin sensitivity in STZ group in this experiment.

Previous Type 1 diabetes studies on rats and humans showed lower plasma IGF-1 levels, consistent with findings in the present study [45,46]. Reduced IGF-1 levels may be associated with increased blood glucose and decreased insulin levels. Ghrelin injection on the left-ventricular dysfunction animal model suppressed cardiac cachexia development, while chronic treatment of GHSs increased IGF-1 levels in human subjects [47,48,49]. However, no effect of Hex was observed on plasma IGF-1 in the present study, indicating that Hex may not exert an effect on circulating IGF-1. The treatment time in this experiment may not have be long enough to alter plasma IGF-1 levels, as previous studies used longer treatment times. In addition to unchanged IGF-1, STZ-induced rats exhibited significantly elevated levels of FFAs relative to whole-body adipose tissue weight. Increased FFA levels have been reported in blood plasma and various tissues because of impaired glucose metabolism in diabetes [50,51,52]. A Type 2 diabetes study exhibited that reduced glucose uptake and utilization, predominantly in muscle tissue, were correlated with increased FFA concentration [6]. Glucose oxidation was depressed by elevated FFA oxidation through inhibition of glycolytic enzymes, resulting in suppressed glycogen synthesis. A further study demonstrated that body glucose uptake decreased by 30–40% in diabetic patients, compared to non-diabetic subjects [53]. Furthermore, an anti-lipolytic drug study showed reduced FFA levels via improved glucose metabolism in diabetic rats [54]. The present study exhibited recovered levels of plasma FFAs following Hex treatment. Hex has been shown to reduce blood glucose levels through normalization of islet β-cell function and stimulation of insulin secretion in a previous study [40]. Therefore, normalized plasma FFA levels may be a consequence of improved glucose metabolism.

In conclusion, Hex is capable of recovering impaired pulsatile GH secretion through increasing hypothalamic GHRH activity in STZ-induced diabetes, suggesting a possible mechanism in improving metabolic balance in diabetes. Hex may recover β-cells from STZ-toxic effect and increase insulin sensitivity to improve plasma glucose levels and glucose-stimulated insulin secretion. Therefore, Hex may serve as a drug in the treatment of diabetes to recover normal insulin and GH profiles.

## 4. Materials and Methods

### 4.1. Diabetic Rat Model

Male Wistar rats (150–200 g, 6 weeks of age) were used in the study. Animals were randomly divided into control and STZ groups. Rats in the STZ group received a single dose of freshly prepared STZ (65 mg/kg) intra-peritoneal injection, while animals in the control group were given the same dosage of sodium citrate buffer, as previously described [55]. Three days post-STZ injection, blood glucose level was measured by Accu-Chek glucometer (Roche, Indianapolis, IN, USA). Fasting blood glucose over 16.6 mmol/L was considered as diabetes. Hex treatment (100 μg/kg) was commenced after 4 weeks of diabetes induction and injected intra-peritoneally for 2 weeks. All animal experiments conformed to the Guide for the Care and Use of Laboratory Animals published by the Australian National Health and Medical Research Council and was approved by the Animal Ethics Committees of The University of Queensland (AE02316, March 2013).

### 4.2. Blood Collection

The blood correction method has been previously described [56]. Animals had free access to food and water through blood collection. During diabetes development, animals were trained to be familiar with procedure of sampling in order to avoid stress generated by consecutive blood collection. On the day of experiment, 36 sequential tail-tip blood samples were collected from 8:00 am at 10 min intervals for 6 h. A 1 mm portion of distal tail was excised by surgical blade and a small volume of blood was pooled on the tip of tail. Four microliters of whole blood were collected using a 10 μL Gibson pipette and transferred into a 500 μL Eppendorf tube which contained 116 μL of 0.05% PBS-T. Samples were then mixed by vortex and placed on dry ice. After collection, a gentle pressure was applied on the wound to stem blood flow. Because of repeat sampling, the original wound may had been disrupted, and the tail wound gently immersed in physiological saline (0.9% sodium chloride; Baxter, Old Toongabbie, New South Wales, Australia) and if necessary, the remaining scab was removed by using the edge of surgical blade. After blood collection was finished, samples were transferred to a −80 °C freezer for storage until further analysis.

### 4.3. Analysis of Pulsatile Growth Hormone Secretion by Sensitive Sandwich ELISA

GH concentration was determined by a sensitive sandwich ELISA which was previously described [56]. Briefly, a 96-well plate was coated by monkey anti-rat antibody (1:40,000, NIDDK–NHPP, Torrance, CA, USA) and incubated overnight at 4 °C. Blocking buffer (5% skim milk powder in 0.05% PBS with Tween-20) was then applied on the plate to reduce non-specific binding. Standard cure was generated by rat GH. In general, rat GH was 1:2 serially diluted in PBS-T with BSA (0.2% PBS-T/BSA). GH standards and samples were then bounded by 50 μL detecting antibody (rabbit anti-serum to rat GH antibody, 1:40,000, NIDDK-NHPP, Torrance, CA, USA). Fifty microliters of anti-rabbit IgG (1:2000, GE Healthcare Ltd., Little Chalfont, Bucks, UK) were added to bound complex after detection antibody incubation. One-hundred microliters of *O*-phenylenediamine (OPD, Invitrogen, Carlsbad, CA, USA) were applied to each well for enzymatic colorimetric reaction. Reaction was stopped by adding 50 μL 3M HCl to each well. The plate was read at a 490-nm wavelength on a Rainbow 96 monochromatic microplate reader (Tecan Trading AG, Männedorf, Switzerland). GH concentration was calculated by regression of the standard curve.

### 4.4. Deconvolution Analysis

Deconvolution analysis was featured for pulsatile GH secretion kinetics and secretary patterns [57,58]. Parameters, such as basal, pulsatile, total GH secretion, and the mode of secretary bursts (time in minutes from pulse onset to the peak of the burst), were included in the measurements. The orderliness of regularity of serial GH serum concentration was calculated by approximate entropy analysis [59].

### 4.5. Glucose Tolerance Test (GTT) and Insulin Tolerance Test (ITT)

Following Hex treatment, GTT was conducted after overnight fasting. Forty microliters of blood samples were collected from the animals’ tail veins at time 0, followed by an intraperitoneal glucose injection at 2 g/kg body weight. Subsequently, the same amount of blood samples was collected at 15, 30, 45, 60, 90, and 120 min points. Blood plasma samples were then collected through centrifugation at 6000 rpm for 3 min at room temperature and stored in a −80 °C freezer for insulin and glucose measurements. Three days following GTT, ITT was performed after 6 hours fasting. Human recombinant insulin (1 IU/kg, Novo Nordisk, Demark) was intraperitoneally injected and blood glucose was measured at time 0, 15, 30, 45, 60, 90 and 120 min points using an Accu-Chek glucometer.

### 4.6. Euthanization

Animals were anesthetized with sodium pentobarbital (100 mg/kg body weight) after GTT and ITT. Cardiac puncture was conducted with a heparinized syringe (100 IU/mL) for blood sample collection. Blood samples were immediately centrifuged (6000 rpm for 3 min at room temperature) and plasma were stored in −80 °C freezer for insulin, IGF-1 and FFAs measurements.

### 4.7. Glucose and Hormone Analysis

Blood glucose level was determined by Glucose Colorimetric Assay kit (Cayman Chemical, Ann Arbor, MI, USA) and collected during GTT. Because of the higher glucose concentration in diabetic animals, samples from STZ and STZ + Hex groups were diluted 1:20, while 1:5 dilution was applied to control and control + Hex groups. Circulating levels of IGF-1, insulin, and FFAs were determined by Mouse/Rat IGF-1 Quantikine ELISA kit (R&D Systems, Minneapolis, MN, USA), Mouse/Rat Insulin ELISA kit (EMD Millipore, St. Charles, MI, USA), and nonesterified fatty acids (NEFA-C) Assay (Wako, Osaka, Japan), respectively. All procedures were performed according to the manufacturer’s instructions.

### 4.8. Statistical Analysis

All data were expressed as mean ± S.E.M. One-way ANOVA with Tukey post-hoc test was carried out for multiple comparisons, as appropriate. In all comparisons, the differences were considered to be statistically significant at a value of *p* < 0.05.
